# Impacts of food contact chemicals on human health: a consensus statement

**DOI:** 10.1186/s12940-020-0572-5

**Published:** 2020-03-03

**Authors:** Jane Muncke, Anna-Maria Andersson, Thomas Backhaus, Justin M. Boucher, Bethanie Carney Almroth, Arturo Castillo Castillo, Jonathan Chevrier, Barbara A. Demeneix, Jorge A. Emmanuel, Jean-Baptiste Fini, David Gee, Birgit Geueke, Ksenia Groh, Jerrold J. Heindel, Jane Houlihan, Christopher D. Kassotis, Carol F. Kwiatkowski, Lisa Y. Lefferts, Maricel V. Maffini, Olwenn V. Martin, John Peterson Myers, Angel Nadal, Cristina Nerin, Katherine E. Pelch, Seth Rojello Fernández, Robert M. Sargis, Ana M. Soto, Leonardo Trasande, Laura N. Vandenberg, Martin Wagner, Changqing Wu, R. Thomas Zoeller, Martin Scheringer

**Affiliations:** 1 Food Packaging Forum Foundation, Zurich, Switzerland; 2 Department of Growth and Reproduction, International Center for Research and Research Training in Endocrine Disruption of Male Reproduction and Child Health (EDMaRC), Rigshospitalet, University of Copenhagen, Copenhagen, Denmark; 3 Department of Biological and Environmental Sciences, University of Gothenburg, Gothenburg, Sweden; 4 Institute of Biogeochemistry and Pollutant Dynamics, ETH Zurich, Zurich, Switzerland; 5 Centre for Environmental Policy, Imperial College London, London, UK; 6 Department of Epidemiology, Biostatistics and Occupational Health, Faculty of Medicine, McGill University, Montreal, QC Canada; 7 Department Adaptation du Vivant, Unité mixte de recherche 7221, CNRS (French National Research Center) and Muséum National d’Histoire Naturelle, Paris, France; 8 Institute of Environmental & Marine Sciences, Silliman University, Dumaguete, Philippines; 9 Institute of Environment, Health and Societies, Brunel University, Uxbridge, UK; 10 Healthy Environment and Endocrine Disruptor Strategies, Commonweal, Bolinas, CA USA; 11 Healthy Babies Bright Futures, Charlottesville, V.A., USA; 12 Nicholas School of the Environment, Duke University, Durham, NC USA; 13 The Endocrine Disruption Exchange, Eckert, CO USA; 14 Center for Science in the Public Interest, Washington, DC USA; 15 Independent Consultant, Frederick, MD USA; 16 Institute for the Environment, Health and Societies, Brunel University London, Uxbridge, UK; 17 Environmental Health Sciences, Charlottesville, Virginia USA; 18 Department of Chemistry, Carnegie, Mellon University, Pittsburgh, PA USA; 19 IDiBE and CIBERDEM, Universitas Miguel Hernandez, Elche, Spain; 20 University of Zaragoza, I3A, Zaragoza, Spain; 21 Green Science Policy Institute, Berkeley, CA USA; 22 Division of Endocrinology, Diabetes, and Metabolism, Department of Medicine, University of Illinois at Chicago, Chicago, IL USA; 23 Department of Immunology, Tufts University School of Medicine, Boston, MA USA; 24 Department of Pediatrics, NYU Grossman School of Medicine, New York, NY USA; 25 Department of Environmental Health Sciences, School of Public Health & Health Sciences, University of Massachusetts Amherst, Amherst, MA USA; 26 Department of Biology, Norwegian University of Science and Technology (NTNU), Trondheim, Norway; 27 Department of Animal and Food Sciences, University of Delaware, Newark, DE USA; 28 Department of Biology, University of Massachusetts Amherst, Amherst, MA USA; 29 RECETOX, Masaryk University, Brno, Czech Republic

**Keywords:** Food contact material, Migration, Food packaging, Non-intentionally added substance, Chronic disease, Human health, Mixture toxicity, Endocrine disrupting chemical, Sustainable packaging, Circular economy

## Abstract

Food packaging is of high societal value because it conserves and protects food, makes food transportable and conveys information to consumers. It is also relevant for marketing, which is of economic significance. Other types of food contact articles, such as storage containers, processing equipment and filling lines, are also important for food production and food supply. Food contact articles are made up of one or multiple different food contact materials and consist of food contact chemicals. However, food contact chemicals transfer from all types of food contact materials and articles into food and, consequently, are taken up by humans. Here we highlight topics of concern based on scientific findings showing that food contact materials and articles are a relevant exposure pathway for known hazardous substances as well as for a plethora of toxicologically uncharacterized chemicals, both intentionally and non-intentionally added. We describe areas of certainty, like the fact that chemicals migrate from food contact articles into food, and uncertainty, for example unidentified chemicals migrating into food. Current safety assessment of food contact chemicals is ineffective at protecting human health. In addition, society is striving for waste reduction with a focus on food packaging. As a result, solutions are being developed toward reuse, recycling or alternative (non-plastic) materials. However, the critical aspect of chemical safety is often ignored. Developing solutions for improving the safety of food contact chemicals and for tackling the circular economy must include current scientific knowledge. This cannot be done in isolation but must include all relevant experts and stakeholders. Therefore, we provide an overview of areas of concern and related activities that will improve the safety of food contact articles and support a circular economy. Our aim is to initiate a broader discussion involving scientists with relevant expertise but not currently working on food contact materials, and decision makers and influencers addressing single-use food packaging due to environmental concerns. Ultimately, we aim to support science-based decision making in the interest of improving public health. Notably, reducing exposure to hazardous food contact chemicals contributes to the prevention of associated chronic diseases in the human population.

## Background

We, as scientists working on developmental biology, endocrinology, epidemiology, toxicology, and environmental and public health, are concerned that public health is currently insufficiently protected from harmful exposures to food contact chemicals (FCCs). Importantly, exposures to harmful FCCs are avoidable. Therefore, we consider it our responsibility to bring this issue to the attention of fellow scientists with relevant expertise, but currently not engaged in the area of FCMs, as well as decision makers and influencers in government, industry and civil society dealing with environmental and health-related aspects of food packaging. We propose that a broader, multi-stakeholder dialogue is initiated on this topic and that the issue of chemical safety of food packaging becomes a central aspect in the discussions on sustainable packaging.

Food contact chemicals (FCCs) are the chemical constituents of food contact materials and finished food contact articles, including food packaging, food storage containers, food processing equipment, and kitchen- and tableware [1, 2]. We define FCCs as all the chemical species present in food contact articles, regardless of whether they are intentionally added or present for other reasons.

It is clearly established by empirical data that FCCs can migrate from food contact materials and articles into food, indicating a high probability that a large majority of the human population is exposed to some or many of these chemicals [3]. Indeed, for some FCCs there is evidence for human exposure from biomonitoring [4–11], although some FCCs may have multiple uses and also non-food contact exposure pathways.

When food contact material regulations were first developed, it had been generally assumed that low-level chemical exposures, i.e. exposures below the toxicologically established no-effect level, pose negligible risks to consumers, except for carcinogens [12, 13]. However, more recent scientific information demonstrates that this assumption is not generally valid, with the available evidence showing that exposure to low levels of endocrine disrupting chemicals can contribute to adverse health effects [14–20]. In addition, chemical mixtures can play a role in the development of adverse effects [21–24], and human exposure to chemical mixtures is the norm but currently not considered when assessing health impacts of FCCs [1]. The timing of exposures during fetal and child development is another critical aspect for understanding development of chronic disease [25]. Currently, these new and important insights are still insufficiently considered in the risk assessment of chemicals in general, and of FCCs in particular [20]. We have previously published an in-depth analysis of the scientific shortcomings of the current chemical risk assessment for food contact materials in Europe and the US [1]. For example, in the European Union (EU) the regulation EU 10/2011 includes a list of authorized substances for the manufacture of plastic materials and articles in contact with food and, for some of the chemicals, their permitted maximum concentration, either in the plastic food contact article or in food (i.e. specific migration limit) [26]. However, there are still many substances that are present in plastics and other materials as non-intentionally added substances (NIAS). Even though the EU regulations 10/2011 explicitly and EU 1935/2004 generally require a risk assessment of NIAS, there are many difficulties: first, identification of NIAS is very demanding [27] and, secondly, studying the effects on human health is often not possible because for example the chemicals are not available as pure substances or testing would be too expensive [1]. What is more, there is no regulatory requirement to assess toxic effects of the chemical mixtures migrating from food contact articles [1]. To summarize, we are concerned that current chemical risk assessment for food contact chemicals does not sufficiently protect public health.

Therefore, we would like to bring the following statement to the attention of policy makers and stakeholders, especially those currently working on the issue of packaging waste but not focusing on the chemical safety of food contact articles (Table 1). By mapping the challenges (Table 2), we aim to initiate a broader debate that also involves scientists with different expertise of relevance to the issue. Importantly, chemical safety must be addressed in two ways: e.g. (i) a discussion of how chemical safety is ensured, based on the current scientific understanding and e.g. (ii) a debate of the chemical safety of food packaging in the circular economy, which aims at minimizing waste, energy and resources use [28]. Therefore, we provide an overview of the most pressing challenges based on current scientific understanding. Ultimately, the public is to be protected from exposures to hazardous FCCs while at the same time the aims of the circular economy need to be achieved. To reach these goals, we think that there is a need to better inform decision making on future food packaging research and policy.

**Table 1:** Overview of relevant stakeholders from the food contact and circular economy domains. Inter-governmental organizations could convene these stakeholders from different backgrounds and initiate topical discussions on the issues detailed in Table 2

Stakeholder group	Description
Intergovernmental organization	Staff and expert working groups of the World Health Organization, Food and Agriculture Organization, United Nations Environment Programme, etc.
Regulatory	Global government officials and regulatory authority experts in the areas of food contact and circular economy
Enforcement	Enforcement officers
Risk assessment	Experts in government agencies, third-party labs, industry and international working groups
Packaging and product design	Experts designing and developing new “sustainable packaging” or business models for food products in circular economies
Global food production	Multinational food (processing) industry experts and decision makers
Local food production	Farmers and primary producers, hospitality sector representatives
Retail	Decision makers and experts on distribution of locally and globally produced foods
Food packaging manufacturing	Chemical manufacturers (polymers, additives), converters, packaging manufacturers and their supply chains
Food contact article manufacturing	Chemical manufacturers, food processing equipment manufacturers, kitchen- and tableware manufacturers, other food contact article producers and their supply chains
Waste management	Government officials, industry experts and providers
Civil society	Environmental and health NGOs, consumer advocacy groups, food movements
Science	Academics, researchers in industry, governments, and NGOs, independent scientific consultants

**Table 2:** Topics of concern (based on Table 2 [1]) and examples of activities addressing them. This is not a complete and comprehensive overview but rather a starting point for further discussions that essentially need to involve many stakeholders (see Table 1)

Area	Topic	Description	Example
A. DATA GAPS	1. Information on chemicals used in food contact materials	Characterize types of chemicals used in the manufacture of FCMs and FCAs, their functions and levels	[67]
	2. Information on non-intentionally added substances	Compile existing information, develop strategies and work plans to fill data gaps	[139, 140]
	3. Information on migration of food contact chemicals	Provide systematic overview of evidence for migration from FCMs and FCAs	[130]
	4. Empirical exposure data	Measure migration into actual foods, assess intake for different demographics (age groups, ethnic and regional diversity)	
B. METHODOLOGY GAPS AND NEEDS	5. Comprehensive definition of adverse effects	Expand the scope of toxicological testing requirements to include non-cancer related endpoints such as effects on the nervous, immune and endocrine systems, and cardiovascular and metabolic effects	
	6. Approaches to addressing non-monotonic dose response	Develop practical tools for use in chemical risk assessment of FCCs	[119]
	7. Approaches to addressing mixture toxicity	Develop overall migrate testing for finished FCAs that can be used in the regulatory context, including standardized sample preparation	[141]
	8. Develop a framework to address aggregate exposures	Integrate exposure information from different legislative areas when setting safe exposure thresholds	[142]
	9. Develop a framework to address cumulative exposures	Assess the safety of exposures to different chemicals through the same or different exposure routes	
	10. Modernize tiered approach for screening and prioritization	Include additional relevant endpoints for toxicity testing, include testing of finished FCA	
	11. Compile information on human health outcomes of exposure to FCCs	Assess systematically the available evidence for how FCCs adversely impact human health; highlight data gaps showing the need for appropriate longitudinal studies that assess food contact chemicals	[130]
C. UPDATE REGULATORY PROCESSES	12. Overall regulatory framework for evaluation beyond sector-specific regulations	Combine chemical hazard and possibly risk assessment for different sectors in one legal framework	
	13. Requirements for data on use of FCCs	Based on the principles of REACH, set legal requirement to provide information about chemical use for market access	
	14. Need to reassess substances authorized for use and/or generally recognized as safe	Policy instruments for removing authorized chemicals e.g. indirect food additives, EU starting substances and additives for plastic FCMs	
	15. Address bias in risk assessment	Ensure that scientific judgement is placed in context of personal values, acknowledge other sources of bias and balance expert groups accordingly	
	16. Ensure transparency of decisions	Communicate potential or real bias of decision makers and experts making recommendations for decision makers	
	17. Improve enforcement	Raise awareness to provide resources for enforcement authorities to expand activities	[136]
	18. Multi-stakeholder dialogues on practical solutions	Address two key topics: 1.) Definition of safety for FCCs: update according to current scientific knowledge; 2.)Food packaging in the circular economy: chemical safety considerations	
	19. Integrate food packaging waste and safety considerations	Policy must address both aspects simultaneously to avoid conflicting goals	[138]
D. REPLACING HAZARDOUS FCCs	20. Developing safer alternatives	Based on revised definition of safety and updated toxicity testing; develop screening assays for endocrine disruption and other relevant endpoints	[134, 143]
	21. Testing finished food contact articles	Use combination of toxicity testing and chemical analysis (“Effect-directed analysis”) to screen for hazardous but unknown FCCs	[95]
	22. Integrating human health with environmental considerations: life cycle approach	Develop integrative assessment for environmental and human health impacts, e.g. using life cycle analysis or other method	[144]
	23. Update sustainable packaging concept	Define sustainable packaging to also include aspects of human health protection that are based on current scientific understanding	

## Part 1. Facts based on established scientific data and findings

### Chemicals migrate from all types of food contact materials and articles

Chemicals can transfer from food contact materials and articles into food. This phenomenon is known as *migration* and has been studied since the 1950s [29–33]. All types of food contact materials may exhibit chemical migration, but the types of migrating chemicals and their levels differ significantly. There are around 1200 peer-reviewed scientific studies clearly demonstrating migration of multiple FCCs from food contact materials and articles (for example [29–32, 34–66]). Migration is affected by temperature, storage time, the chemistry of both the food contact article and food, the thickness of the food contact layer, and the packaging size (proportionally higher migration from smaller packaging sizes due to the increasing surface-area-to-volume ratio).

### Several thousands of chemicals are intentionally used to make food contact articles

Analysis of FCC lists issued by legislatures, industry, and NGOs worldwide indicates that almost 12,000 distinct chemicals may be used in the manufacture of food contact materials and articles [67]. For example, European Union (EU) and EU Member State regulations list a total of 8030 substances for use in different types of food contact articles [68]. In the United States (US), 10,787 substances are allowed as direct or indirect food additives, and roughly half of these are FCCs [69]. Many additional FCCs may be used in the US under the assumption of being generally recognized as safe (GRAS), but they are not notified to the US Food and Drug Administration (FDA) and therefore no public record on their use is available [70]. In general, information on the actual use of a chemical in food contact materials (and its levels) is difficult to obtain [71, 72].

### Toxicity and exposure information is available only for few of the intentionally used chemicals

All migrating FCCs have inherent toxicity properties that can cause different effects at different doses and are related to the timing of exposure, mode of action, and other aspects. At the same time, levels of FCCs that humans are exposed to reflect their use (or presence) in a food contact article and are associated with its concentration in food. To evaluate the risk of a given chemical to human health, information on its inherent toxicity (i.e., its hazard) and the actual levels of exposure is needed.

Many of the chemicals that are intentionally used in the manufacture of food contact articles have not been tested for hazard properties at all, or the available toxicity data are limited [67]. Moreover, endocrine disruption, as a specific hazard of concern, is not routinely assessed for chemicals migrating from food contact articles, although some chemical migrants are known endocrine disruptors [73–77].

Exposure data are commonly based on assumptions or estimates – for example derived from dietary assessments or unpublished (proprietary) data of an intentionally used FCC’s concentration in a food contact article [71, 78, 79]. Thus, there is significant uncertainty associated with these data. In short, decisions on the use of a chemical in food contact materials are commonly made in data-poor situations.

### Known hazardous chemicals are authorized for use in food contact

Substances of very high concern (SVHC) are defined under the EU regulation on the Registration, Evaluation, Authorisation and Restriction of Chemicals (REACH) as chemicals with unacceptable hazard properties (like carcinogenicity, mutagenicity, toxicity for reproduction, persistence and bioaccumulation, or endocrine disruption). The human health effects of chemicals used in the manufacture of food contact materials are not covered by REACH. However, the toxicity properties of SVHCs are identical regardless of their use. Several SVHCs (i.e., known hazardous chemicals) are authorized for use in food contact in Europe and other countries [80]. For several SVHCs, as well as other known hazardous substances, there is evidence for migration from food contact articles [73], for example migration of several ortho-phthalates [81], per- and polyfluoroalkyl substances [63, 82–84], and perchlorate [85]. Notably, for substances classified as SVHCs there is a societal consensus in Europe that their use shall be phased out. Furthermore, in a circular economy, where food packaging is made from recycled materials, it is essential to ensure that no hazardous chemicals are present in the materials because it will be very difficult, if not impossible, to manage their risks effectively.

### Food contact articles contain non-intentionally added substances (NIAS) and most are unknown

In addition to intentionally used chemicals, food contact articles also contain non-intentionally added substances (NIAS) that may or may not have a technical function. NIAS are impurities of starting substances or additives, reaction by-products generated during manufacture along the entire supply chain, or break-down products, e.g. from additives [86]. More and more NIAS, including those with evidence for migration, have been detected by modern analytical methods, but many remain unidentified due to prevailing limitations in structure elucidation [27, 55, 74, 87–95]. However, it is well known that for several types of food contact materials, migration of NIAS is more significant than migration of intentionally used substances [3, 96]. In conclusion, there are many unknown and/or untested chemicals present in food contact articles.

### Risk assessment of unknown chemicals is not possible under the current regulatory approach

All migrating FCCs need to be assessed for their risk to human health, and this requires information on both hazard and exposure levels. But these data cannot be generated for chemicals with unknown identity. Therefore, the conventional risk assessment approach cannot be applied to assessing the safety of these unidentified FCCs [1]. Moreover, these NIAS contribute to the mixture of migrating chemicals, which likewise cannot be evaluated by conventional risk assessment strategies. This implies that the human population is exposed to unknown and/or untested chemicals migrating from food contact articles, with unknown health implications.

### Humans are exposed daily to mixtures of chemicals migrating from food contact articles into food

A chemical mixture can cause adverse effects even if all individual components of the mixture are present at individually safe levels. Mixture toxicity is a scientifically well-described phenomenon [23]. In general, FCCs migrating from food contact articles are assessed for their safety on a substance-by-substance basis. However, chemical migration occurs in mixtures, therefore humans are also exposed to chemical mixtures [1]. In addition to chemicals migrating from food contact articles, humans are exposed to chemicals from other sources, such as personal care products, food contaminants or textiles.

## Part 2. Areas of uncertainty

### FCCs from food contact articles are a significant source of chemical exposure

In the US, 40,655 chemicals are used in commerce today [97] and in Europe 22,169 substances have been registered under REACH [98]. Human exposure has been systematically assessed for a fraction of these chemicals, including for some FCCs [4, 99–105]. However, for the majority of FCCs it remains unknown if humans are exposed and at what levels. At least 3221 exogenous chemicals have been measured in human blood [106]. Various xenobiotics have frequently been found in pregnant women, in the placenta and cord blood, indicating that fetal exposure to mixtures of xenobiotics is the norm [107–111], and the health impacts of this mixture exposure during early life remain largely unknown, but effects on the human brain (i.e. decreased intelligence in children that were exposed prenatally to a mixture of EDCs) have been shown [21]).

Approximately 12,000 intentionally added substances [67] and 30,000 to 100,000 non-intentionally added substances potentially migrate into food from various food contact articles [112]. Appropriate chemical-analytical methods are lacking for most of them [27, 90, 95, 113, 114], which makes it currently impossible to discern the contribution of these chemicals to the overall human exposure to xenobiotics. However, food packaging is estimated to be the most relevant source of human exposure to plasticizers [71, 115]. For comparison: it is well known that the concentrations of FCCs in foodstuffs exceed the concentrations of e.g. pesticide residues in food by at least a factor of 100 [3]. Moreover, there are fewer than 1000 pesticides in commercial use, and chemical-analytical methods are available for all of them, including their main metabolites.

### Using generic thresholds for the safety assessment of FCCs is inadequate

For FCCs without hazard data, generic concentration thresholds are commonly used in their risk assessment. For example, in Europe unauthorized chemicals may be used in food contact plastics if their migration into food is below the detection limit of 10 ppb (10 μg/kg food), and if they are not genotoxic, mutagenic, toxic to reproduction, or substances in nano-form [26]. In practice this detection limit is often interpreted as a safety threshold, for example in the revised Japanese FCM legislation [116]. In the US, 0.5 ppb (0.5 μg/kg food) is the Threshold of Regulation: for FCCs migrating below this threshold, no hazard data need to be provided if the chemical’s structure has no alerts for genotoxicity [117]. But this approach is inadequate because it assumes that “the dose makes the poison”. According to the current scientific understanding of non-monotonic dose responses, this is clearly not always the case [18, 19]. Deriving a safe threshold may not be straightforward for chemicals with non-monotonic dose responses, such as for endocrine disrupting chemicals [14], because the results of standard high-dose toxicity testing cannot simply be extrapolated to the low-dose range. Chemicals with non-monotonic dose responses not only have increasing effects with increasing dose but may also have effects in the low-dose range [15, 19]. A study commissioned by the European Food Safety Authority concluded that non-monotonic dose responses for several food-related chemicals (such as FCCs) could neither be confirmed nor rejected, and further investigation has been recommended [118]. Guidance for addressing this difficulty in chemical risk assessment has been published [119]. Therefore, we find it concerning that this aspect is not being taken up in general by (regulatory) chemical risk assessment as it implies that humans are unnecessarily exposed to hazardous chemicals.

### Humans may be exposed to FCCs also from sources that are non-food contact articles

Exposure levels to a given FCC need to be known to determine if there is a risk, as a chemical’s risk is related to both exposure and hazard. But in practice, obtaining exposure data for FCCs is difficult, as the European research project FACET has demonstrated [78], because there is no systematic surveillance. As a result, exposure levels of FCCs are mostly based on estimates, which are associated with uncertainty and potentially underestimate actual risk [79].

In addition, humans can be exposed to an FCC from non-food contact sources (e.g. bisphenol A from food packaging and thermal-paper receipts), too. This implies that aggregate exposure is probable for at least some FCCs [101, 120]. Therefore, a threshold approach based on exposure from only a single source may lead to an underestimation of actual human risk since the actual exposure is underestimated.

### Combined exposure to mixtures of FCCs increases risk to human health

Combination effects are not adequately addressed, even though it is known that chemicals migrate from food contact articles in mixtures. Novel approaches, such as effect-directed analysis, where in vitro toxicity testing is combined with chemical analysis, or semiquantitative assessments, where chemicals with assumed highest exposure levels are identified, can be useful for prioritization of analytical chemistry work to identify chemicals of concern in mixtures [27, 74–76, 86, 92, 121]. In vitro screening for some aspects of endocrine disruption can be used to assess such hazard properties in the overall migrate, but the sample preparation, assay selection and data interpretation aspects need to be further developed [75, 95, 122–124].

### The consequences of human exposure to FCCs need careful investigation

The duration of time between FCC exposure and health impact in humans can be very long, and suitable longitudinal studies in the human population are mostly lacking [125]. Therefore, linking FCC exposure to adverse human health outcomes is very difficult for many relevant substances.

Furthermore, in epidemiology, exposures to FCCs are often not routinely assessed [126] and only studied for very few individual FCCs. For some of the most controversial chemicals like bisphenol A and phthalates, evidence continues to accumulate while thousands of other FCCs that migrate into food lack hazard and/or exposure information [127–129]. Therefore, a systematic assessment of the available evidence is required [130] in order to identify and address pertinent knowledge gaps. This is of urgency also for economic reasons, as the additional disease costs related to exposures to endocrine disrupting chemicals are significant, with annually US$ 340 billion in the US and US$ 217 billion in the EU [131].

## Part 3. Options for improvement

There is clear scientific evidence that chemicals migrate from food contact articles, and it is likely that the majority of the human population is affected by these exposures. Some of the migrating chemicals are known hazardous substances. Establishing causality between chronic human exposure to chemicals from food packaging (or other types of food contact articles) and adverse health outcomes in humans is difficult. As a consequence, there is a potentially large, but essentially unquantified, burden of risk currently placed on citizens who are unknowingly ingesting mixtures of unidentified and untested chemicals that originate from food contact articles with their daily food.

In addition, food packaging is of special interest in discussions around the circular economy, and many solutions are currently being proposed for reuse, recycling or replacing plastic food packaging with alternative materials. But those solutions mostly focus only on single aspects such as reducing CO_2_ emissions, energy use or plastic littering, and often omit considerations of chemical safety. This may lead to regrettable substitutions that cause problems later.

Therefore, we urge policy makers, regulators, food and food packaging manufacturers, civil society and scientists from within the FCM world, and outside, to address more attention to the issue of assessing the safety of food contact chemicals, as it appears to be an important opportunity for prevention of chronic diseases associated with hazardous chemical exposures. We identify seven areas of highest concern where we see an urgent need for discussion and improvement:

### Eliminating hazardous chemicals in food contact articles

Known hazardous chemicals should not be used in the manufacture of food contact articles if their presence in the finished article, by means of modern chemical analysis, cannot be excluded to a reasonable extent. Some business operators have taken action on their food packaging in the US [132], but other food contact articles, such as processing equipment or filling lines, need to be considered as well, and requirements should become legally binding to encompass all food contact articles. Authorized lists of chemicals for food contact uses should be revised and known hazardous chemicals removed, such as substances of very high concern (SVHC), if their use is considered non-essential [133].

### Developing safer alternatives

If the use of known hazardous chemicals is essential and currently no suitable substitutes are available, research into developing safer alternatives should be a priority. The development of safer alternatives should be based on current scientific principles, such as the Tiered Protocol for Endocrine Disruption (TiPED) [134], as the safety definitions and related criteria for establishing the risk of FCCs for human health currently in place in the EU, US and elsewhere are not aligned with the latest scientific understanding [1]. Importantly, hazard identification and characterization should be performed prior to large-scale FCC use.

### Modernizing risk assessment

Together with industry and civil society, regulatory agencies should update what hazard and exposure data are necessary for making safety determinations, based on current scientific understanding [1]. Authorized substances for food contact that are currently in use should be reassessed accordingly; this will also require a transparent prioritization strategy that determines which chemicals are reassessed first. Any values-based or expert decisions affecting, for example, exposure estimates, should be made transparent, e.g. when dealing with data gaps or scientific uncertainty [135].

### Including endocrine disruption

Chemical hazards related to endocrine disruption should be assessed for all chemicals migrating from food contact articles and for all substances that are intentionally used in their manufacture. It is important to consider the non-monotonic dose response phenomenon in chemical risk assessment [135].

### Addressing mixture toxicity

The mixture toxicity of the overall migrate, i.e. all chemicals migrating from food contact articles, should be determined for a relevant set of hazards such as genotoxicity, mutagenicity, and endocrine disruption. This means that finished food contact articles should be tested in addition to single chemicals intentionally used in their manufacture. Regulators and other stakeholders should invest in research and development for fast or high-throughput approaches to screen the overall migrate. A general mixture toxicity uncertainty factor should be introduced until better approaches for dealing with mixtures have been developed.

### Improving enforcement

Importantly, new regulations must be enforceable, and sufficient resources for compliance control must be made available to authorities [136]. Enforcement of rules for known hazardous chemicals, such as carcinogens, is necessary. Food contact articles should not be a source of carcinogens migrating into food [128, 129, 137]. Enforcement of rules on mixture toxicity must also be practically feasible.

### Finding practical solutions

A multi-stakeholder dialogue should be established to identify solutions that are sustainable and focus on the same goal, namely protecting humans and the environment while providing effective, efficient and affordable food packaging in a circular economy. Such solutions may be global in scope, but stakeholder needs will likely vary between countries and cultural regions, and such needs must be taken into account. Importantly, the interface between food packaging and waste management should be considered when new, practical solutions are developed [138].

In Table 2, we provide an overview of different topics that are relevant for the areas of concern.

## Conclusions

We highlight that the human population is exposed via food to chemicals migrating from food contact articles such as food packaging. Many of these chemicals are not sufficiently assessed for their impacts on human health, while others are known hazardous substances. As a consequence, we see a need for revising how the safety of migrating chemicals is assessed, using current scientific understanding. At the same time, different stakeholders are pushing for solutions to reduce packaging waste and end plastic pollution, but oftentimes not taking chemical safety into consideration. Therefore, we encourage all stakeholders to focus more on this issue and employ science-based decision making in the interest of improving public health. Reducing exposure to hazardous food contact chemicals contributes to the prevention of associated diseases in humans. And including chemical safety considerations in the development of sustainable packaging will lead to solutions that are beneficial to both human and environmental health.

## Data Availability

Not applicable.

## Abbreviations

FCC: Food contact chemical
NIAS: Non-intentionally added substance
REACH: Registration, Evaluation, Authorisation and Restriction of Chemicals
SVHC: Substance of Very High Concern
TiPED: Tiered protocol for endocrine disruption
